# Plasticity of the *Leishmania* genome leading to gene copy number variations and drug resistance

**DOI:** 10.12688/f1000research.9218.1

**Published:** 2016-09-20

**Authors:** Marie-Claude N. Laffitte, Philippe Leprohon, Barbara Papadopoulou, Marc Ouellette

**Affiliations:** 1 Centre de Recherche en Infectiologie du Centre de Recherche du CHU Québec, and Département de Microbiologie, Infectiologie et Immunologie, Faculté de Médecine, Université Laval, Québec, Québec, Canada

**Keywords:** Leishmania, Ploidy, Drug Resistance, Mode of action, Cos-Seq

## Abstract

*Leishmania *has a plastic genome, and drug pressure can select for gene copy number variation (CNV). CNVs can apply either to whole chromosomes, leading to aneuploidy, or to specific genomic regions. For the latter, the amplification of chromosomal regions occurs at the level of homologous direct or inverted repeated sequences leading to extrachromosomal circular or linear amplified DNAs. This ability of *Leishmania* to respond to drug pressure by CNVs has led to the development of genomic screens such as Cos-Seq, which has the potential of expediting the discovery of drug targets for novel promising drug candidates.

## Introduction

*Leishmania* are dimorphic parasites living as extracellular promastigotes in the digestive tract of *Phlebotomus* or *Lutzomyia* sandflies and as intracellular amastigotes within phagocytic cells (mainly macrophages) of the vertebrate hosts. *Leishmania* species cause leishmaniasis, the second largest parasite killer; there are 1.3 million new cases annually, 12 million people are affected worldwide, and 350 million people are currently at risk ^1^. The *Leishmania* genus includes several species, among which more than 20 are pathogenic to humans ^2^. *Leishmania* can be divided into two subgenera: the *Leishmania Leishmania* subgenus responsible for visceral and cutaneous leishmaniasis and the *Leishmania Viannia* subgenus often associated with either cutaneous or muco-cutaneous forms of the disease ^2^. Visceral leishmaniasis is mainly caused by *L. donovani* and *L. infantum* and is characterized by fever, hepatosplenomegaly, and pancytopenia ^3^, making it the most severe and deadly form of the disease when compared with the self-healing but nonetheless debilitating skin lesions of cutaneous leishmaniasis ^4, 5^.

No effective human vaccine is currently available against *Leishmania* (a canine *Leishmania* vaccine was recently registered in Europe ^6^), and control measures mainly involve chemotherapy ^7– 9^. Pentavalent antimony (Sb) has been the standard drug for 70 years and remains the mainstay in many endemic regions, apart from Northern India, where antimonial formulations have been rendered obsolete because of widespread parasite resistance. Other first-line therapies include the polyene antibiotic amphotericin B (AMB) for which a single dose was shown to be 95% effective against visceral leishmaniasis in India ^10^. Liposomal AMB has become a standard treatment in many countries ^11^ but requires administration by the intravenous route. Geographical differences in the response to liposomal AMB were reported, and visceral leishmaniasis cases in India were more responsive than those from East Africa or South America ^12^. Clinical AMB resistance is scarce and parasites remained susceptible even after multiple rounds of treatment in the same patient ^13^. The alkyl-lysophospholipid analogue miltefosine (MTF) is the only oral drug against *Leishmania* ^14, 15^. It has been successfully used for the treatment of visceral leishmaniasis since its registration in 2002 in India ^16^. However, relapse rates are on the rise in India ^17, 18^ and Nepal ^19, 20^, making MTF resistance a likely problem. The aminoglycoside paromomycin (PMM) is also approved for the treatment of visceral leishmaniasis in India ^21, 22^. So far, the scarce use of PMM has limited the emergence of resistance, but geographical variations in PMM efficacy against visceral leishmaniasis were noted between East Africa (especially Sudan) and India ^23, 24^. Lastly, pentamidine (PTD) has been abandoned for the treatment of visceral leishmaniasis because of serious toxicities and is mainly restricted to patients with cutaneous leishmaniasis in South America ^25– 27^.

Despite six decades of use, the mode of action (MOA) of antimonials is not known. It has been shown to lead to the production of reactive oxygen species ^28– 31^, the depletion of trypanothione ^32^, and apoptosis-like death ^33– 36^, but an exact MOA is still awaited. The same applies for MTF, AMB, PTD, and PMM, with the possible exception of AMB, which kills *Leishmania* by forming pores in ergosterol-containing membranes. New molecules with well-defined drug targets are clearly needed.

## *Leishmania* and its genome

The *Leishmania* genome is around 32 Mb and displays over 8,300 coding genes ^37, 38^. Within the *Leishmania* genus, gene synteny is conserved for more than 99% of genes between *L. major*, *L. infantum*, and *L. braziliensis*, and only few species-specific genes were found ^38^. *Leishmania* species have between 34 and 36 chromosomes ranging in size from 0.3 to 2.8 Mb ^37– 41^. One unique feature characterizing trypanosomatid parasites lies in their genome architecture, their protein-coding genes being organized as large polycistronic units ^42, 43^. In the absence of defined RNA polymerase II promoters, transcription of the long polycistronic units occurs in a bidirectional fashion from transcriptional start sites located at strand switch regions ^43, 44^. Processing into individual messenger RNAs (mRNAs) occurs by the addition through *trans*-splicing of a spliced leader RNA (39 nt) to the 5′ ends of each mRNA, coupled to 3′ end polyadenylation ^45, 46^. Because of its lack of transcriptional control, *Leishmania* uses several adaptive mechanisms to regulate gene expression when facing changing environmental conditions during its development. 3′ untranslated regions (3′ UTRs) were shown to be major players in monitoring mRNA stability and translation rates in this parasite ^47– 53^. To overcome stressful conditions like drug pressure, *Leishmania* also often relies on DNA copy number variations (CNVs) (aneuploidy, gene amplification, or gene deletion) for regulating the expression of drug targets, drug transporters, or other determinants of resistance. This is not restricted to *Leishmania*, however, and variations in gene dosage or chromosome copy numbers also influence drug susceptibility, adaptability, and proliferation in fungi and cancer cells ^54– 57^. In addition to CNVs, single-nucleotide polymorphisms (SNPs) in drug targets or in transporters can lead to drug resistance without the need for altering gene expression.

## Copy number variations

During the last few decades, *Leishmania* parasites were considered to be essentially diploid but recent data have shown that aneuploidy seems to be the norm ^58– 64^. Within populations of *Leishmania* parasites, distinct aneuploidy patterns were shown to occur at the level of individual cells. This phenomenon was called mosaic aneuploidy and can translate into a seemingly average diploid population when the cumulative ploidy is derived from next-generation DNA sequencing data but in which few parasites actually share the same ploidy for individual chromosomes ^62, 63, 65^. Interestingly, variations in the size and content of chromosomes have also been observed between different strains of the related trypanosomatid parasite *Trypanosoma cruzi* ^66, 67^. In the case of *Leishmania*, circumstantial links between the presence of supernumerary chromosomes or chromosomal losses and drug resistance have been observed ^58– 60, 64, 68– 71^, suggesting that a particular group of genes on the variant chromosomes may possibly act together in establishing resistance, but this has yet to be demonstrated.

Aneuploidy is generally linked to developmental abnormalities as best exemplified by the trisomy 21 syndrome in humans. However, *Leishmania* uses aneuploidy as a lifestyle. This is raising a number of questions about aneuploidy generation, stability, transmission, and biological significance (reviewed elsewhere ^60, 62, 72^). In the absence of transcription initiation control, increases (or decreases) in chromosome copy number may serve as a strategy for regulating expression under environmental cues. This can happen at the level of whole chromosomes, and indeed there was a good correlation between chromosome ploidy and the level of DNA and RNA expression ^59^. Increasing the copy numbers of entire chromosomes may lead to the overexpression of toxic genes, but the *Leishmania* genome (32 Mb) is spread in 34 to 36 chromosomes, thus reducing the co-expression of many genes. However, as explained in detail below, *Leishmania* also has the ability to amplify (or delete) specific smaller regions of DNA as part of extrachromosomal elements by recombination/rearrangements at the level of homologous repeated sequences (RSs). RNA levels derived from these amplifications are correlated to DNA copy number.

The genome of *Leishmania* is populated with repeated DNA sequences. A recent study highlighted the entire set of RSs in different *Leishmania* species, and it was found that the whole *Leishmania* genome has the potential to be rearranged at the level of those RSs for generating extrachromosomal elements ^73^. Indeed, almost 2,000 RSs are distributed over the genome of *L. infantum* and these potentially support the formation of more than 3,000 extrachromosomal DNA elements ^73^. Short interspersed degenerate retroposons (SIDERs) account for up to 65% of all RSs. SIDERs are truncated versions (~0.55 kb) of formerly active retroposons that are predominantly located in 3′ UTRs and have been associated with post-transcriptional regulation at the levels of both mRNA stability and translation ^47, 48, 52, 53, 74, 75^. Because SIDERs are degenerated, they were found in different RS groups. Remarkably, SIDER elements would have dual roles: one functional by regulating gene expression and a second one structural, providing the backbone to facilitate gene rearrangements for changing copy number of chromosomal DNA regions.

**Figure 1.**
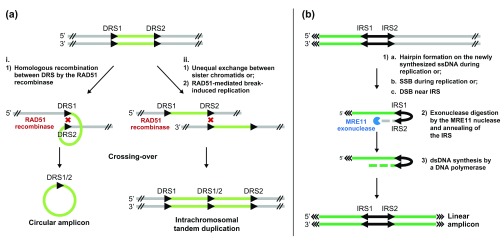
Potential mechanisms for gene amplification in *Leishmania*. ( **a**) The RAD51 recombinase mediates homologous recombination between direct repeated sequences (DRS) and leads to (i) extrachromosomal circular amplicon or (ii) intrachromosomal tandem duplication by unequal sister chromatid exchange or RAD51-mediated break-induced replication. Black arrows represent DRS. ( **b**) The MRE11 nuclease processes DNA ends after single-strand break (SSB), double-strand break (DSB), or hairpin formation during replication and leads to extrachromosomal linear amplification. Black arrows represent inverted repeated sequences (IRS). The green segments represent the amplified DNA regions. dsDNA, double-stranded DNA; ssDNA, single-stranded DNA.

Extrachromosomal DNA amplifications are frequently detected in *Leishmania* parasites challenged with drugs or other stressful conditions ^58, 59, 64, 76– 87^. The episomes are amplified as either circular or linear extrachromosomal DNA and formed through rearrangements at the level of direct or inverted homologous RSs, respectively ( Figure 1) ^73, 80, 88^. Interestingly, between 60% and 80% of the predicted amplicons appear to be already present in the population in the absence of selection and these pre-existing stochastic gene amplifications were shown to foster the selection of adaptive traits in response to drug pressure ^73^. Beneficial amplicons were shown to increase in abundance upon higher drug pressure and to decrease when the drug is removed, allowing parasites to respond to a changing environment ^73^.

Since gene rearrangements through RSs are primary responses to drug pressure, a reasonable hypothesis was that identifying recombinase proteins involved in these rearrangements could lead to strategies to prevent the emergence of resistance. A first candidate was the RAD51 DNA repair protein, a key protein involved in homologous recombination (HR), a mechanism evolutionarily conserved in trypanosomatids ^89^. Interestingly, the expression of RAD51 was induced in *Leishmania* by DNA double-strand breaks (DSBs) ^90, 91^. Inactivating RAD51 led to viable parasites unable to generate circular extrachromosomal elements but still capable of producing linear amplicons upon drug pressure ^73^. *Leishmania* has three RAD51 paralogs (RAD51-3, RAD51-4, and RAD51-6) that were shown to work as a complex in promoting HR through their capacity to stimulate RAD51 activity ^92^. Inactivation of RAD51-4 was also shown to prevent the formation of circular amplicons in *L. infantum* exposed to drugs, but not of linear amplicons ^92^.

Linear amplicons are formed by the annealing of RSs found in an inverted orientation ^64, 73, 93^. MRE11 is a DNA repair nuclease that interacts with RAD50 and NBS1 to form the MRN complex ^94, 95^ and is important for DSB repair by HR ^96, 97^ or for non-homologous end joining ^89^ ( Figure 1b). Inactivation of MRE11 impaired the ability of *L. infantum* to form linear amplicons upon drug selection at the level of inverted RSs, although the capacity to generate circular amplicons was similar to that of wild-type parasites ^93^. Moreover, a fully functional MRE11 is important for linear amplification, as parasites expressing DNA-binding-proficient but nuclease-deficient MRE11 exclusively generated circular amplicons during drug selection ^93^. Interestingly, inactivation of MRE11 alone or along with its partner ** RAD50 led to extensive chromosomal translocation in *L. infantum* ^98^, showing that the MRE11/RAD50 complex is important for the maintenance of genome integrity in addition to its role in gene rearrangements. The number of enzymes involved in the formation of extrachromosomal elements suggests that targeting this pathway may not be a viable strategy for preventing the emergence of resistance, although this remains to be experimentally tested.

## Single-nucleotide polymorphisms and small nucleotide insertions or deletions

Although CNVs are important contributors of drug resistance, SNPs and small nucleotide insertions or deletions (indels) can also contribute to resistance. This was proven with experimental drugs ^99, 100^ and was highlighted with MTF where amino acid substitutions or non-sense mutations were observed in the MTF transporter (MT) ^101^ or in its Ros3 subunit ^102^. This was further confirmed in additional mutants using whole genome sequencing ^103, 104^ or by deep sequencing of MT ^105^. Mutations detected in the MT gene of *L. infantum* isolates serially collected from an MTF-treated patient who had multiple relapses were shown to correlate with resistance ^106^, suggesting that MTF resistance could become a clinical reality in the near future.

Genome-wide surveys of genetic variations in *L. donovani* isolates from the Indian subcontinent supported the notion that resistance to antimonials emerged on several distinct occasions ^58, 107^. Isolates could also be clustered on the basis of their genetic structure and haplotypes, with some groups being enriched for non-responsive strains ^58, 107, 108^. Interestingly, a particular group of highly resistant isolates that clustered together were found to share genomic features associated with resistance ^107^. Among these were a higher copy number for the H-locus, coding for the well-characterized ABC transporter MRPA ^109^, and a homozygous two-base-pair insertion in the aquaglyceroporin 1 (AQP1) gene involved in Sb uptake and whose inactivation or downregulation is strongly correlated with resistance ^71, 107, 110– 117^. The potential for these genomic variants in predicting treatment outcome is exciting, given the lack of molecular markers for Sb resistance, but will need to be thoroughly evaluated by using larger and geographically diversified sets of well-defined isolates.

## Exploiting copy number variations for understanding drug mode of action and resistance mechanisms

Target-based assays and phenotypic whole-cell-based assays are the cornerstones of drug discovery. The current trend for anti-parasitic agents is for whole cell assays. A drawback of phenotype-based assays is the lack of knowledge about the targets of hit compounds. Although the molecules could be brought to the clinic without further knowledge about their MOA, a clear understanding of the molecular targets will facilitate the improvement of a candidate drug through lead optimization. Characterization of drug-resistant mutants, which often revealed mutations or CNVs in drug targets or in proteins responsible for drug transport, is one strategy to pinpoint drug targets. However, it is salient to point out that this strategy has not yet led to targets against the current anti-leishmanials, although amplification of gene targets was observed in mutants made resistant to experimental drugs ^64, 99^. Since CNVs are often associated with resistance, forward genetic tools can experimentally mimic this. One such gain-of-function screen was based on functional cloning where *Leishmania* cosmid libraries were electroporated into *Leishmania* and these transfectants were selected for a specific phenotype ^118^. Selection is possible because of the high copy number (and gene expression) of the cosmids. This screen was successfully applied while selecting for drug resistance or susceptibility ^101, 114, 119– 123^. This technique selects for cosmids conferring dominant phenotypes (leaving out less enriched cosmids) and is not easily amenable to high-throughput screening. The sensitivity of cosmid-based functional screening was enhanced by its recent coupling to next-generation sequencing in an approach termed Cos-Seq ^124^. The proportion of parasites with cosmids providing a selective advantage is expected to rise with increasing drug pressure, and these can be tracked and quantified at each drug increment by Illumina sequencing ^124, 125^. Thus, the dynamics of cosmid enrichment can be followed over the entire course of selection instead of being monitored only at endpoint. Published or ongoing Cos-Seq screens using experimental drugs with known targets (for example, methotrexate, terbinafine, and 5-fluorouracil) confirmed the recovery of the relevant target genes by Cos-Seq ^124^. Interestingly, Cos-Seq supported the hypothesis that the current anti-leishmanials (MF, AMB, Sb, PMM, and PTD) may not act via specific major protein targets ^124^. Indeed, although an unprecedented number of resistance genes (known and novel) were isolated using Cos-Seq, none emerged as a clear target candidate and it is conceivable that these antiquated drugs are broadly cytotoxic by disrupting multiple minor targets. Whether some of the genes are genuine drug targets remains to be established, and non-protein targets represent another possibility as these would not be detected by Cos-Seq.

The advent of high-content screening for intracellular *L. donovani* amastigotes ^126^ is also key in the search for novel molecules having favourable anti-leishmanial properties directly on the intracellular stage of the parasite. This allowed the discovery of 192 new leads against visceral leishmaniasis from an initial set of 1.8 million compounds from GlaxoSmithKline ^127^. An MOA could be hypothesized for 80 of the lead compounds using prior proprietary knowledge and bioinformatics analyses of TriTryp genomes, which revealed an over-representation of putative kinase inhibitors ^127^. Cos-Seq was initially carried out with the insect form of the parasite but this could easily be adapted to intracellular parasites and this technique could be used to find the targets of these promising novel molecules or of other drugs repurposed against *Leishmania* ^128^.

The Cos-Seq technique does not allow the isolation of loss-of-function mutations such as those found in the aquaglyceroporin AQP1 or in the MT transporter genes (see above). These require high-throughput dominant negative screening approaches, like inducible RNA interference target sequencing (RIT-Seq), which proved instrumental in elucidating mechanisms of drug uptake in trypanosomes ^129^. Although RNA interference is absent from the *L. Leishmania* subgenus, it is active in species of the *L. Viannia* subgenus ^130^. The lack of inducible expression in *Leishmania* was also a limitation of this technique, but two recent reports have shown the feasibility of inducible expression in *Leishmania* ^131, 132^. Thus, it is theoretically possible to develop a technology similar to RIT-Seq in *L. Viannia* parasites. An alternative approach to RIT-Seq would be to rely on RNA-guided nuclease systems using clustered regularly interspaced short palindromic repeats (CRISPR) and CRISPR-associated (Cas) enzymes, as these have proven very efficient for achieving targeted genomic modifications in a wide range of genomes ^133– 135^. In trypanosomatid parasites, the CRISPR/Cas9 system derived from *Streptococcus pyogenes* has been used for disrupting genes in *L. major* ^136^, *L. donovani* ^137^, and *T. cruzi* ^138^ and in principle could be used for generating whole-genome Cas9-mediated gene deletion libraries.

## Concluding remarks

The toolkit for drug target discovery and resistance mechanism elucidation for *Leishmania* is expanding. With new promising drug candidates in the pipeline and further technological developments, it should now be possible to find new targets which should further help in the control of this important neglected tropical disease.

## Abbreviations

3′ UTR, 3′ untranslated region; AMB, amphotericin B; AQP1, aquaglyceroporin 1; Cas, clustered regularly interspaced short palindromic repeat-associated; CNV, copy number variation; CRISPR, clustered regularly interspaced short palindromic repeat; DSB, double-strand break; HR, homologous recombination; MOA, mode of action; mRNA, messenger RNA; MT, miltefosine transporter; MTF, miltefosine; PMM, paromomycin; PTD, pentamidine; RIT-Seq, RNA interference target sequencing; RS, repeated sequence; Sb, antimony; SIDER, short interspersed degenerate retroposon; SNP, single-nucleotide polymorphism.
